# Client satisfaction and associated factors towards the health service provided to members of a community-based health insurance scheme in Southern Ethiopia

**DOI:** 10.3389/frhs.2023.1237895

**Published:** 2023-11-03

**Authors:** Getachew Ossabo Babore, Taye Mezigebu Ashine, Asnakech Zekiwos Heliso, Teshome Tesfaye Habebo

**Affiliations:** ^1^ Department of Comprehensive Nursing, School of Nursing, College of Medicine and Health Science, Wachemo University, Hossana, Ethiopia; ^2^ Department of Disease Prevention and Promotion, Kembeta Tembaro Zone Health Department, SNNP, Hossana, Ethiopia

**Keywords:** community-based health insurance, client satisfaction, enrolled, health facilities, Ethiopia

## Abstract

**Background:**

Globally, 1.3 billion poor people have no access to health services due to their inability to afford payment when they need services. According to a report published by the WHO in 2014, globally 150 million people are pushed into poverty as a result of direct payment for health services.

**Objective:**

This study aims to assess the satisfaction level of clients and associated factors toward health services provided to members of a community-based health insurance (CBHI) scheme.

**Methods:**

An institutional-based cross-sectional study design was employed. A total sample size of 393 people was estimated using a single population formula, and three health facilities (HFs) were selected using a simple random sampling method, whereas study participants were selected by using a systematic sampling method. All patients who visited the HFs were included, whereas women who visited the HFs for maternity service were excluded from the study. A reliability test (Cronbach’s alpha) was performed to determine the internal consistency for these items to measure the satisfaction level of the clients. Epi Info software version 7 was used to calculate the sample size and to enter data, whereas further data cleaning and analysis were conducted using SPSS software version 20.

**Results:**

A total of 367 clients enrolled in the community-based health insurance scheme were interviewed, showing a response rate of 93%. The reliability test (Cronbach's alpha) value for the items used to measure level of client satisfaction was 0.817. The overall level of the clients’ mean satisfaction toward health service provision was 63.1% (3.95 + 0.47 SD). This study found that age with AOR = 0.11 [95% CI (0.01–0.79)], residence with AOR = 1.80 [95% CI (1.79–3.66)], number of family with AOR = 2.27 [95% CI (1.46–11.22)], frequency of visits to HFs with AOR = 13.62 [95% CI (2.09–88.58)], and clients’ level of knowledge with AOR = 3.33 [95% CI (1.06–10.42) had a statistical significant association with client satisfaction toward health service provision.

**Conclusion:**

Our study found that the perceived level of client satisfaction is higher than previous studies. Residence, frequency of visits, level of knowledge, payment during referral time, number of family members, and frequency of visits were identified as predictors of client satisfaction on the health service provision.

## Introduction

Health insurance (HI) is one of the nine components of the healthcare financing (HCF) mechanism, which was launched with an ultimate goal of improving health service provision (1). Moreover, it is a key factor in averting the financial hardship associated with paying for health services, which is comprehended through implementing the three HCF sub-functions: revenue collection, pooling of resources, and purchasing of services (2, 3). In the contemporary period, the increasing demand for medical care is contributing to the increasing healthcare costs. Subsequently, it is becoming a real barrier to the affordability and availability of the services (4, 5). However, HI became an emerging tool to guarantee the health of the families. Hence, once the families are enrolled in the scheme, they will have peace of mind, they will have no delay in seeking medical care when they become sick, and they will be free from predicting what their medical bill will be (5).

Under the umbrella of HI, community-based health insurance (CBHI) is an emerged opportunity for the pro-poor communities (6). Thus, it is a not-for-profit type of insurance and stands against the cost during seeking medical treatment for illness as well as reduces out-of-pocket (OOP) expenditure for healthcare (6–8). The CBHI scheme is a good alternative for the impoverished community. It must stand alone based on the premises of risk-pooling and community solidarity. In addition, it is characterized by a feature of volunteer membership and trust enrollment (9, 10). Eventually, this plays a critical role in attaining sustainable fully functioning universal health coverage (UHC) (11, 12).

However, different types of HI, CBHI, and social health insurance (SHI) are common in Ethiopia. They are potential strategies recognized as an instrument to finance healthcare by mobilizing resources for the citizens in the formal and small informal sectors, respectively (13). In Africa, the first initiative of CBHI began under the direction of emigrant workers, and it commenced in 1986 in the Democratic Republic of Congo (14). In Ethiopia, CBHI was introduced in 2010 and piloted within 13 districts after 1 year, those selected from four major regions (Amhara, Oromia, SNNPR, and Tigray) (15, 16).

Under CBHI, satisfaction of the enrollees is the desired outcome of healthcare services. Moreover, client satisfaction reflects the gap between the expected services and the experience of the service (17, 18). In fact, satisfaction influences a person who seeks medical advice and adherence to the treatment and assures continuous attractiveness of the service contracted. Furthermore, it is a fundamental tool that reflects how well the healthcare system is working (19, 20). Hence, it is an important indicator to predict the retention tendency of enrollees in the scheme (21).

Poor HCF has been seen as a major challenge for the healthcare system, and it leaves households (HHs) vulnerable to the impoverished from catastrophic health expenditure (22). Globally, 1.3 billion poor people have no access to health services due to their inability to afford the payment at the time. Consequently, they are subjected to financial hardship (23). According to a report published by the World Health Organization (WHO) in 2014, globally 150 million people suffered a financial catastrophe; out of them, 100 million were pushed to poverty as a result of direct payment for health service. Furthermore, they suffer financial shocks each year having unexpected expenditure for expensive emergency care (24–26). Thus, high reliance on OOP expenses leads an individual and families to reduced utilization of healthcare, ultimately worsening health, and enforcing the risk of impoverishment (27). To this end, recent studies conclude that a person is exposed to catastrophic spending, while a patient consumes more than 10% of the annual household income on healthcare. Approximately 12% of the population worldwide spent at least 10% of their HHs’ income to pay for healthcare. For instance, in Latin America, 5% of inhabitants spend more than 40% of their income on medical care per year (28, 29).

**Figure 1:**
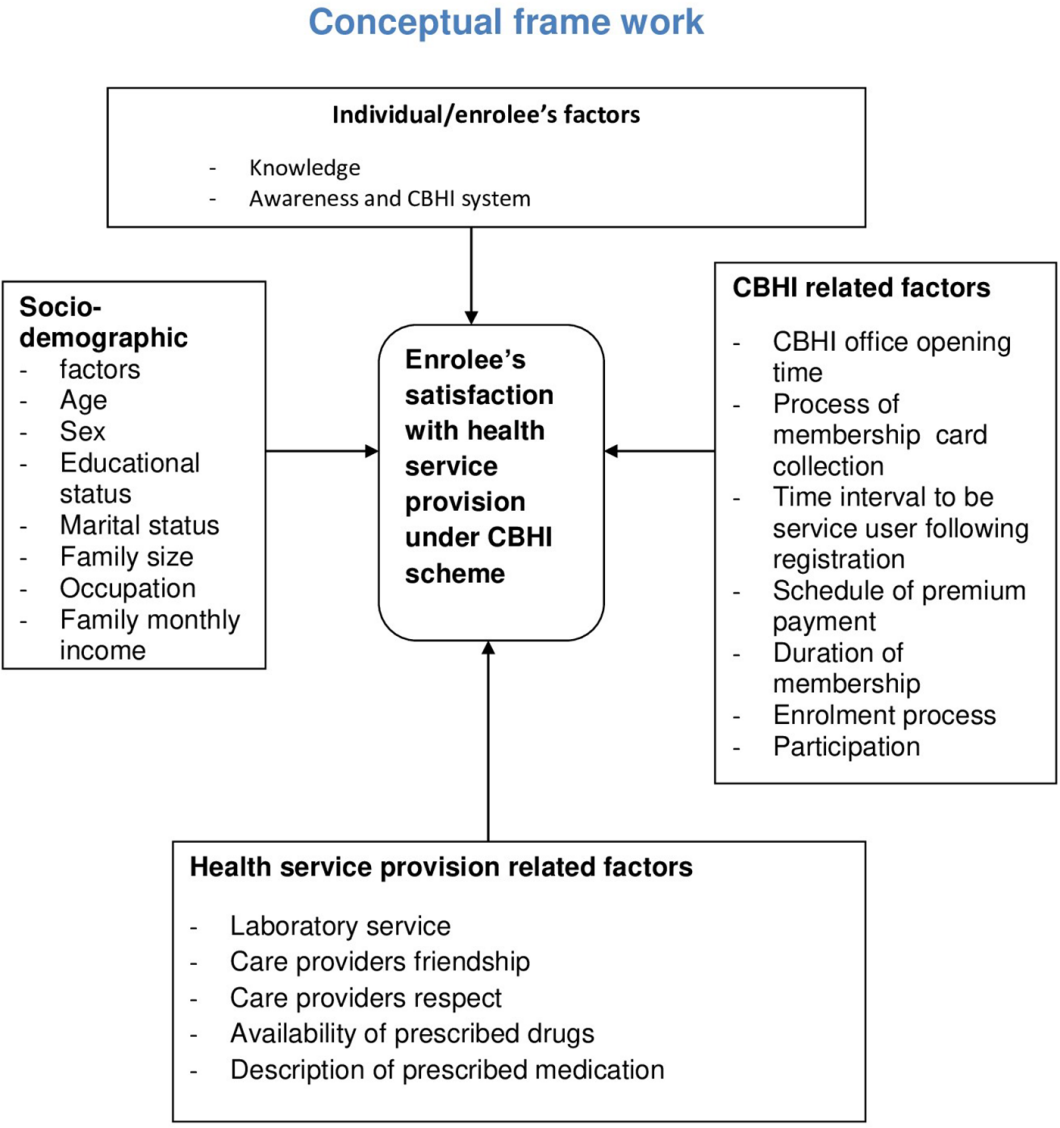
Conceptual framework.

In low- and middle-income countries, more than 35% of health expenses per country come from OOP expenses (30). Moreover, OOP expenses also differ by type and level of income. For instance, in Ghana, Tanzania, and South Africa, it is reported to be 40%, 26%, and 18%, respectively (31). Most low-income countries have to cover medical bills through OOP expenses when they become sick. For instance, in India, Bangladesh, Pakistan, and Ethiopia, it is reported to be 86%, 88.3%, 78%, and 34%, respectively (32). In developing countries, the consequence of OOP for seeking medical care is enormous. More than 2 billion people are affected by inefficiency, inequitable access, and inadequate funding (33). In Ethiopia, HCF mainly relies on foreign donations of approximately 50%, and 33% is covered by OOP payment, whereas the domestic government covers only 17%. Consequently, poor people and communities are always at risk of impoverishment due to expensive medical care. In Ethiopia, having an unpredictable, poorly harmonized donor funding system, low government spending on the health sector financing, and a strong reliance on OOP expenditure result in gaps between aid commitment and actual disbursement. Thus, the cumulative effect of these affects satisfaction of the clients (33–35) (Figure 1).

## Methods

### Study design and setting

An institutional-based cross-sectional study design was employed between 1 July 2019 and 30 September 2019 in Hulbareg woreda, a Silte Administrative Zone in Southern Ethiopia. The Silte Zone is one of the central zones in the SNNPR among 17 zones and seven special woredas. According to the 2017–2018 zonal finance and economic development office census, the projected population of Hulbareg woreda was approximately 82,686. Within the woreda, there are four health centers (HCs). Currently, all HCs were providing services for more than 35,450 enrolled people in the CBHI. For the current physical year (2019), the CBHI coverage was 85%.

### Source and study population

The source population were all household members enrolled in the CBHI scheme and who randomly visited selected health facilities (HFs) for any healthcare. The study population encompassed any member of HHs who visited facilities on the occasion of data collection who was selected by using a systematic sampling method.

### Eligibility criteria

The study included all insured individuals (adults, guardians, or parents of children) who attended outpatient and inpatient departments, whereas members who were insured for less than 6 months and women who visited HFs for maternal service were excluded from the study.

### Sample size determination

The sample size of the study was performed using a single population proportion formula by comparing values obtained from statistical software and manual calculation. The sample size was computed using Epi Info software version 7, by considering the following assumptions: a level of significance of 5% (with a corresponding critical value of *Z_α_*_/2_ = 1.96 for normal distribution), a margin of error of 5%, a proportion (*P*) level of satisfaction on health service provision under CBHI scheme of 42% (17), and taking a total number of 35,450 individuals enrolled in the CBHI scheme. Then, the estimated sample size (*n*) was 371. The sample size for the first specific objective was also calculated by considering the above assumptions using the following statistical formula:

$$\frac{n={\left(Z_{\alpha /2}\right)}^{2}pq}{d^{2}}$$

An estimated sample size after adding a 5% non-response rate was 393. Finally, comparing the estimated sample size in both approaches, a sample size (*n*) of 393 was selected for the study.

### Sampling technique and procedure

To select the study participants, two out of four HFs were selected using the simple random sampling method. Proportionate to population size (PPS) was employed to allocate a sample size for randomly selected HFs (Hulbareg, Ambarcho, and karate). Ahead of the proportional size allocation survey of a total number of clients, who were served during the last 2 months from the facilities, registration books were taken. Then, the estimated sample size (*n*) was divided into the total number of clients (*N*) served during the last 2 months in all selected HFs, which gave a proportionate value (*P*), and by multiplying it with the total served patients, PPS was allocated for each facility. Moreover, the study subjects were selected by using a systematic sampling method, considering the *k*th value (*k* = 2) for each HF. Finally, every *n*th patient was interviewed after completing the treatment at outpatient as well on the discharge date for inpatient.

### Data collection instruments, techniques, and quality management

The structured questionnaire was prepared in English, and it was translated into Amharic and then into the local language Silitigna again. A structured interviewer-administered questionnaire was used to collect data, whereas a tool adapted from different literatures was used in this study. Data were collected by four grade 12 completed students and two nurse supervisors. For items related to satisfaction on CBHI, a five-point Likert scale ordinary response was used to assess the level of client satisfaction. Ahead of the actual data collection to monitor consistency, the protocol of the study tool and client's confidentiality, namely, pre-test, training, and reliability test (Cronbach's alpha), were performed.

### Data processing and statistical analysis

Daily data were cleaned, coded, and then fed into Epi Info version 7. For further cleaning and analysis, the data were transferred into SPSS software version 20. A Likert scale measurement was used to compute the mean score of satisfaction items after extracting questions that fit the reliability test. Model fitness was checked by using Hosmer–Lemeshow goodness-of-fit test, and multicollinearity was also checked by computing the variance inflation factor (VIF). Then, variables had a statistical significance in bivariate analysis with 95% CI at a *p*-value of <0.05, which was considered a candidate variable for the next model. Finally, the statistically significant variables enter into the multivariate regression model to determine the predictors of the satisfaction levels of the clients.

### Ethical consideration

Overall protocol of this study was approved by the Institution Review Board (IRB) of the college of medicine and health science. Ethical clearance and cooperation letters were written for the respective units from the vice president of the research and community service office. Ahead of the interviewer administering question purpose, benefit, and confidentiality issues addressed appropriately, a written informed consent was obtained from each participant.

## Results

### Socio-demographic characteristics

A total of 367 clients enrolled in the CBHI scheme were interviewed, showing a response rate of 93%. The mean age of the respondents was 35.47 (±13.14 SD) years, whereas their age ranged from 19 to 75 years. Among the participants who visited HFs, 209 (56.9%) were male. The mean household size was 5.8, whereas the highest proportion of family size was four and six members per HH, which constituted 74 (20.2%) and 72 (19.6%) of the study, respectively. Annual upper and lower limit membership payment per individual was 100 and 18.18 ETB (Table 1).

**Table 1:** Socio-demographic characteristics of clients who visit the health facilities in Hulbareg woreda, Silte Zone, Southern Ethiopia, 2019.

Variable	Category	Frequency	Percentage
Age of the participants	Less than 24 years	85	23.2
	25–34 years	109	29.7
	35–44 years	81	22.1
	Above 44 years	92	25.1
Sex	Male	209	56.9
	Female	158	43.1
Residence	Rural	273	74.4
	Urban	94	25.6
Educational status	Unable to read and write	160	43.6
	Able to read and write	92	25.1
	Grades 1–8	78	21.3
	Grades 9–12	31	8.4
	College and above	6	1.6
Occupation	Student	54	14.7
	Daily labor	12	3.3
	Merchant	63	17.2
	Household wife	85	23.2
	Farmer	153	41.7
Marital status	Single	86	23.4
	Married	266	72.5
	Widowed/divorced	15	4.1
Membership duration	1–2 years	162	44.1
	More than a year	205	55.9
Monthly income	<500 ETB	197	53.7
	500–1,000 ETB	134	36.5
	<1,000 ETB	36	9.8

ETB, Ethiopian Birr.

### Knowledge of participants on CBHI

**Figure 2:**
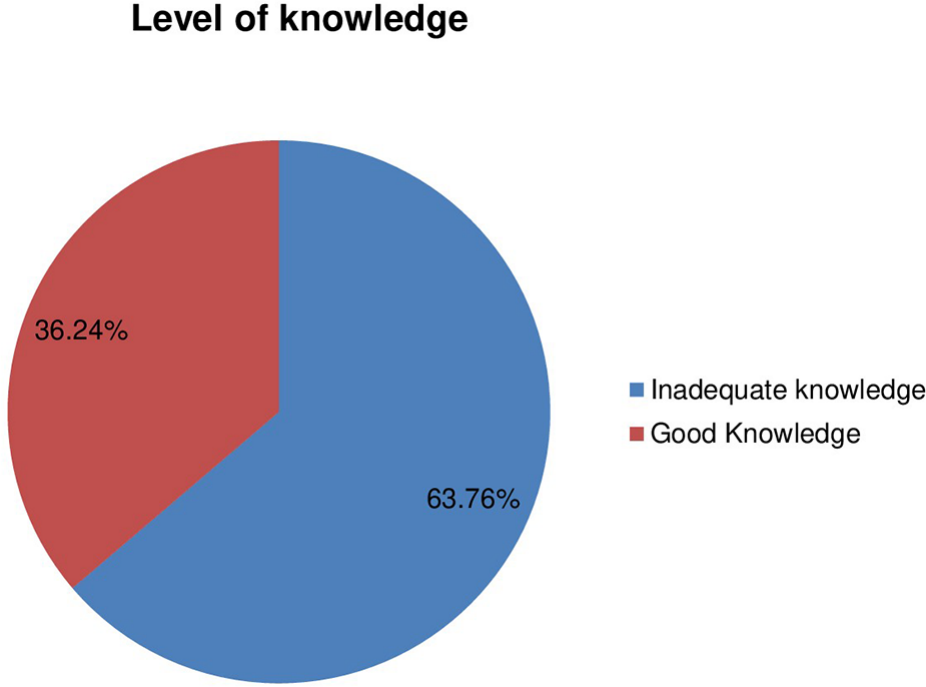
Level of knowledge.

Out of the total participants, 207 (56.7%) have ever heard about CBHI. In addition, the level of knowledge of the clients was assessed by using 13 items. First, the questions were filtered, those that were developed to measure the level of knowledge among participants. Second, the level of knowledge of the individual was assessed by computing the average score of each item. Finally, based on the mean score of the participants, the level of knowledge was categorized as good and inadequate. Accordingly, the majority of the respondents (234, 63.7%) had inadequate knowledge about CBHI (Figure 2)**.**

### Experience of clients with health service provision

**Figure 3:**
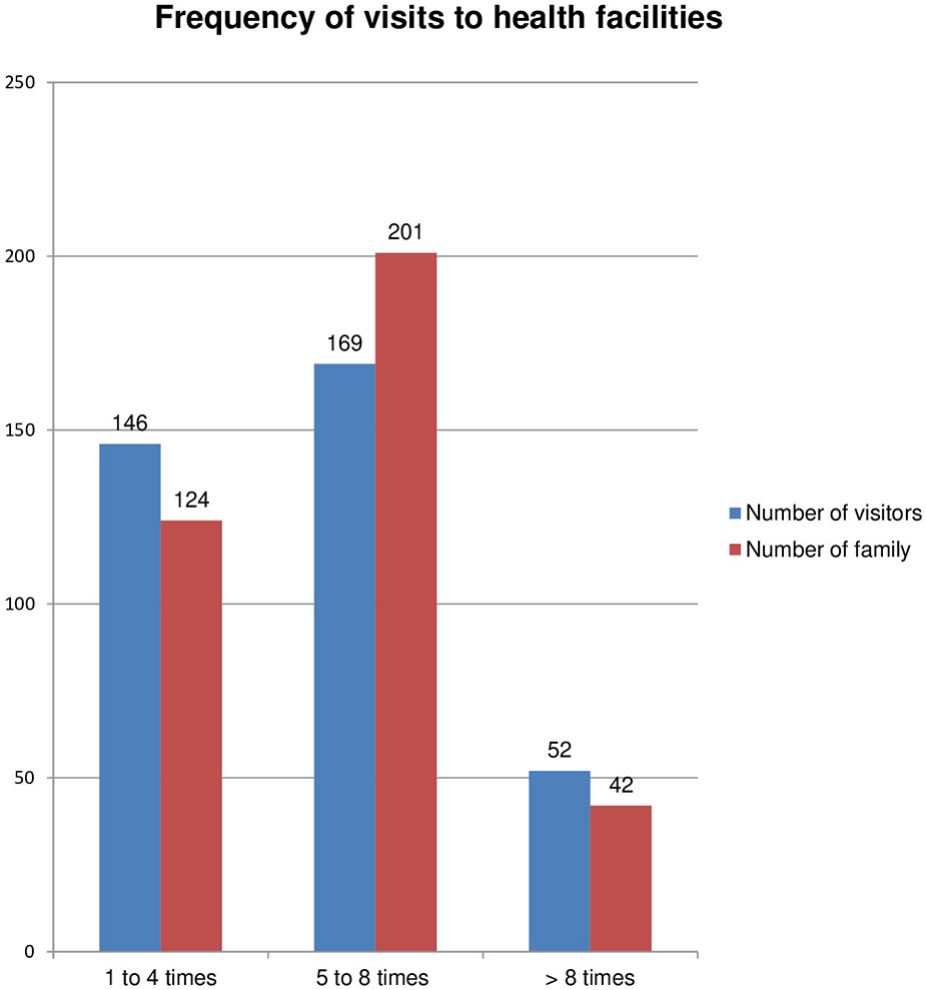
Frequency of visits to HFs.

The average frequency visit to the HF during the last 12 months was 5.67 (±2.38 SD) times. However, the frequency of visits ranged from one to 13 times. More than one-fifth (73.4%) of the respondents visited HF three to five times. A descriptive analysis of the study has shown that the number of families within a single household increased and the number of health-seeking or visits to HFs also increased. More than 50% of the participants witness that the annual premium was very cheap compared with the service offered. An estimated maximum and minimum scale paid for a single visit were 200 and 15.00 ETB, respectively. Moreover, the highest proportion of visitors paid only 15.00 ETB per visit for all the services, which is much lower than the cost of a single antipain (Figure 3)**.**

Among the total participants, only one-third of them had participated in the CBHI insurance concern issue. Moreover, 287 (78.2%) of participants could not get any sensitization training related to CBHI, and the internal or external referral system is one of the packages in the scheme. On top of that, out of them, 130 (36%) clients were referred to other HFs (hospitals), but only 40 (10.9%) were subjected to pay a service charge on the occasion of diagnosis and treatment. The upper and lower payments during the visit were 650 and 150 ETB, respectively.

### Client satisfaction toward health service provision

A five-point Likert scale measurement which ranges from 1 (strongly disagree) to 5( strongly agree) was used to assess client satisfaction toward health service provision. On top of that, to determine the internal consistency of the tools, Cronbach's alpha test was performed first. The items had a score of 0.817.

The possible range of satisfaction for each scale item is 16–80, and they reported the highest satisfaction rate, the services that were received more than the premium with an average score of 4.64 (±0.61 SD), and the services that were available within 24 h with a mean satisfaction rate of 4.48 (±0.75 SD). On the other hand, clients reported poor satisfaction with the item of availability of prescribed drugs (3.26±1.03 SD) and availability of all laboratory tests within the HF (average score 3.32 ± 0.15). The overall level of client mean satisfaction toward health service provision was just above the median score (3.95 ± 0.47 SD). Most of the patients were satisfied on service delivery time over 24 h, and enrollment decreased cost or OOP expenditure for medical bill. The overall level of client (percentage mean score) satisfaction with CBHI was 63.1%. Hence, 56.1% of participants enrolled in CBHI were satisfied with the health service provision (Table 2).

**Table 2:** Level of clients’ satisfaction on health service provision among the members of the community-based health insurance scheme, in Hulbareg woreda, Southern Ethiopia, 2019 (*n* = 367).

Satisfaction assessment questions	Likert scales					Mean score satisfaction SD
	Strongly agree (%)	Agree	Neutral	Disagree	Strongly disagree	
Obtaining service is affordable within 24 h	214 (58.3)	127 (34.6)	17 (4.6)	9 (2.5)	1 (0.2)	4.48 ± 0.75
Healthcare provider intimacy is like as friend for clients	123 (33.5)	196 (53.4)	24 (6.5)	24 (6.5)	0	4.14 ± 0.13
Healthcare providers can provide sufficient information for service	91 (24.7)	201 (60.2)	30 (8.2)	43 (11.7)	2 (0.1)	3.92 ± 0.92
Healthcare providers maintain client confidentiality	109 (29.7)	172 (46.9)	44 (12)	36 (9.8)	6 (1.6)	3.93 ± 0.98
Service delivery areas and setups are comfortable	132 (36.9)	136 (37.1)	52 (14.2)	35 (9.5)	12 (3.2)	3.93 ± 1.09
Enrollment in CBHI helps get better health service	228 (62.1)	93 (25.3)	31 (8.4)	12 (3.3)	3 (0.1)	4.45 ± 0.84
Enrollment increases service utilization	227 (61.8)	82 (22.3)	30 (8.2)	23 (6.2)	5 (1.3)	4.37 ± 0.97
Services that have been obtained are greater than payment/premium	225 (61.3)	95 (25.8)	13 (3.5)	4 (1.1)	0	4.64 ± 0.61
Being a members decrease cost expenditure for medical bill	206 (56.1)	136 (37.1)	15 (4.1)	8 (2.2)	2 (0.1)	4.46 ± 0.73
CBHI office opening time is comfortable to obtain service	146 (39.7)	184 (50.1)	27 (7.4)	7 (1.9)	3 (0.1)	4.26 ± 0.75
All laboratories requested are available in the facility	28 (7.6)	182 (49.6)	71 (19.3)	35 (9.5)	46 (12.5)	3.32 ± 1.15
All prescribed drugs available in dispensary/pharmacy	21 (5.7)	173 (47.1)	73 (19.8)	82 (22.3)	18 (4.9)	3.26 ± 1.023
Time between HF visits and services obtained are average	34 (9.3)	164 (44.6)	80 (21.8)	78 (21.3)	10 (2.7)	3.36 ± 1.01
Healthcare providers can provide adequate time for exam and Rx	39 (10.6)	153 (41.6)	112 (30.5)	57 (15.5)	6 (1.6)	3.44 ± 0.93
HW delivers service respectfully	47 (12.8)	151 (41.1)	107 (29.2)	58 (15.8)	4 (1.1)	3.49 ± 0.94
Membership registration time and service started time comfortable	56 (15.3)	183 (11.4)	88 (5.5)	36 (2.3)	4 (0.3)	3.68 ± 0.89
Overall satisfaction						3.95 ± 0.47

SD, Standard division.

Ordinary regression analysis was performed for the items designed to identify the degree of factors that affects client satisfaction on health service provided under the CBHI scheme. Nearly 70% of respondents responded that they strongly agreed that enrollment in CBHI increased service utilization, while the highest proportion of respondents reported that they strongly disagreed on item availability of ordered tests within the respective facilities (Table 3).

**Table 3:** Ordinary regression for health service-related satisfaction items (*n* = 367).

Variables	Five-point Likert scale client satisfaction	*n* = (%)	Scale	
			Mean ± SD	95% CI
Obtaining service is affordable within 24 h	S. agree	214 (58.3)		
	Agree	125 (34.1)		−1.36 to −0.32
	Neutral	17 (4.6)	4.48 ± 0.75	−4.05 to −1.89
	Disagree	9 (2.5)		−5.39 to −2.47
	S. disagree	2 (0.5)		−7.99 to −.3.24
Service providers act friendly on the occasion	S. agree	123 (33.6)		
	Agree	196 (53.6)		−0.44 to −1.22
	Neutral	23 (6.3)	4.14 ± 0.13	−1.51 to −0.80
	Disagree	24 (6.6)		−2.31 to −1.42
	S. disagree	0		
CBHI office opening time is favorable	S. agree	146 (39.8)		
	Agree	184 (50.1)		−0.95 to 1.71
	Neutral	27 (7.4)	4.27 ± 0.75	−1.82 to −1.04
	Disagree	7 (1.9)		−3.50 to −2.93
	S. disagree	3 (0.8)		
Satisfied with availability of all prescribed drugs	S. agree	21 (5.7)		
	Agree	173 (47.1)		−2.84 to 4.49
	Neutral	73 (19.9)	3.26 ± 1.03	−0.93 to −1.64
	Disagree	82 (22.3)		−0.09 to 0.53
	S. disagree	18 (4.9)		−2.60 to −1.62
Total time from health facility arrival to completion of the treatment	S. agree	34 (9.3)		—
	Agree	164 (44.8)		2.58 to 4.02
	Neutral	80 (21.9)	3.37 ± 1.01	0.71 to 1.38
	Disagree	78 (21.3)		−0.42 to 0.19
	S. disagree	10 (2.7)		−3.40 to −2.12

SD, standard division; S. agree, strong agree; S. disagree, strongly disagree.

### Determinants of client satisfaction

Binary logistic regression at a 5% level of confidence was conducted to select variables for multivariate regression. Among socio-demographic variables, sex is not statistically significant, whereas age, educational status, type of residence, occupation, size of the family, and marital status were statistically significant. Moreover, duration of enrollment, frequency of visits, and payment on the occasion of referral service had a significant statistical association with client satisfaction at *p* < 0.25.

To identify the predictors of client satisfaction, multivariate analysis was performed for the variables that fit the statistical significance on bivariate analysis at *p* < 0.25. This study revealed that age is one of the predictors of client satisfaction level increase by 89.4% (95% CI: 0.01–0.79), members enrolled in CBHI who have resided in rural area are 1.8 times more likely satisfied than urban inhabitants (95% CI: 1.99–3.66). The number of family members in single households increased by one digit clients satisfaction increased by twofold when compared with their counterparties. Clients who were paid a service charge on the occasion of the referral were 79.5% less likely to be satisfied with the service provided compared with those who were not subjected to pay a service charge. When the frequencies of clients' visits to the HFs were increased clients were at greater odds [AOR =  13.62 (95% CI: 2.09–88.58)] of satisfaction as compared with their counterparties and client who had good knowledge of the CBHI scheme increased odds of satisfaction by 3 [AOR = (95% CI: 1.06–10.42)] folds than their counterparties (Table 4).

**Table 4:** Predictors of client satisfaction on health service provision among members of the community-based health insurance, in Hulbareg Woreda, Southern Ethiopia, 2019.

Variable	Category	Level of client satisfaction		COR^a^	95% CI (LL–UL)	AOR 95% CI (LL–UL)
		Dissatisfied	Satisfied			
Age	Less than 24 years	48 (56.5)	37 (43.5)	1		1
	25–34 years	33 (30.3)	76 (69.7)	2.988	1.652–5.402	1.695 (0.233–12.308)
	35–44 years	44 (54.3)	37 45.7)	1.091	0.591–2.012	0.106 (0.014–0.792)^b^
	Above 44 years	36 (39.1)	56 (60.9)	2.018	1.108–3.674	0.566 (0.053–9.311)
Residence	Urban	57 (60.6)	37 (39.4)	1		1
	Rural	104 (38.1)	19 (35.2)	2.503	1.518–4.048	1.801 (1.785–3.658)^b^
Monthly income	<500 ETB	72 (36.5)	125 (63.5)		1	1
	500–1,000 ETB	73 (54.5)	61 (45.5)	0.481	0.308–0.752	1.008 (0.278–3.658)
	>1, 000 ETB	16 (44.4)	20 (55.6)	0.720	0.351–1.477	2.436 (0.351–16.930)
Occupation	Student	35 (64.8)	19 (35.2)	1		1
	Daily labor	5 (41.8)	7 (58.3)	0.354	1.368–0.875	2.565 (0.059–11.545)
	Merchant	39 (61.9)	24 (38.1)	1.234	1.245–8,348	0.977 (0.061–16.281)
	House wife	27 (31.8)	58 (68.2)	3.952	1.923–8.141	2.212 (0.175–27.900)
	Farmer	55 (35.9)	98 (64.8)	3.282	1.716–6.280	2.272 (0.166–31.003)
Educational status	Unable to read and write	48 (30.0)	112 (70.0)	1		1
	Able to read and write	41 (44.6)	51 (55.4)	0.533	0.313–0.908	.598 (0.160–2.229)
	First to eighth grade	41 (52.6)	32 (47.4)	0.387	0.221–0.676	0.600 (0.103–3.512)
	Ninth to 12th grade	25 (80.0)	6 (19.4)	0.103	0.040–0.267	—
	College and above	6 (100	0	—	—	—
Marital status	Single	54 (64.8)	32 (32.2)	1		1
	Married	102 (38.3)	164 (61.7)	2.713	1.647–4.840	3.331 (1.064–0.422)
	Divorced/widowed	5 (33.3)	10 (66.7)	3.375	1.059–10,757	1.591 (0.258–9.831)
Number of family	1–4 family	71 (57.3)	53 (42.7)	1		1
	5–8 family	81 (40.3)	120 (59.7)	1.985	1.261–3.125	2.320 (0.748–7.189)
	More than eight family	9 (21.4)	33 (78.6)	4.912	2.167–11.137	2.274 (1.461–11.218)^b^
Length of membership	1–2 years	89 (54.9)	73 (45.1)	1		1
	>2 years	72 (35.1)	133 (64.9)	2.252	1.477–3.434	1.746 (0.584–5.218)
Number of visits	1–3 visits	26 (59.1)	18 (40.9)	1		
	4–7 visits	88 (37.9)	144 (62.1)	2.364	1.225–4.599	13.619 (2.094–88–583)^b^
	More than eight visits	47 (51.6)	44 (48.4)	1.353	0.653–2.801	4.235 (0.654–27.439)
Knowledge on CBHI issues	Good	123 (52.6	111 (47.4)	1		1
	Poor	38 (28.6)	95 (71.45)	2.770	1.757–4.368	3.331 (1.064–10.422)^b^
Payment on referral service	No	27 (31)	60 (69)	1		1
	Yes	30 (73.2)	11 (26.8)	0.165	0.072–0.377	0.205 (0.063–0.672)^b^

AOR, adjusted odds ratio; COR, crude odds ratio; LL, lower limit; UL, upper limit; CI, confidence interval.

^a^ Statistically significant in bivariate analysis.

^b^ Statistically significant in multivariate analysis.

## Discussion

The study finding revealed that the level of client satisfaction on the health service provided among scheme members was high. The overall mean satisfaction rate was 3.95 ± 0.47 SD with a cumulative percentage mean score of 63.12%. The finding is higher than the studies conducted in Nigeria 42.1%, 46.7% (36, 37), India 42% (38), and 53.3% Istanbul, Turkey (20), whereas it is lower than the study conducted in Bangladesh with a mean score of 4.17 ± 0.04 (39). The higher proportion is possibly attributed to the status of participants. A study conducted in Usman was among the staff, so healthcare providers may have higher expectations toward service provision than other segments of the population, whereas in India, the partial annual premium is covered by the government once the annual payment for healthcare service is covered by the government. Servants may not be questioned for the medical service bill. In this case, this may lead to low satisfaction.

Client satisfaction and their level of knowledge were inversely associated. Participants who had poor knowledge on CBHI were at greater odds of satisfaction with the service provision. The study is in contrast with the study conducted in Nigeria (36). Our finding revealed that 36.3% of participants were knowledgeable about CBHI. According to our study, their level of knowledge toward CBHI was lower than the study conducted in Nepal (89.5%) (40). The possible reason for their low level of knowledge is possibly the difference/composition of study participants. However, their level of knowledge is higher than the study conducted in the Central Zone of Nigeria where only 28.7% (41) of the study population were knowledgeable. High proportion was attributed to the study design and participants. The Nigerian study encompasses both members and non-members of the insurance scheme.

We also found that the level of satisfaction of the participants varied for different satisfaction measurement items. The most client satisfied domain was the services delivered in the respective facilities were available throughout 24 h (4.68 ± 0.76 SD) and that clients strongly agreed on healthcare workers who act friendly while serving them. In turn, friendliness of the service providers increases client satisfaction by 33.6%; this finding is supported by a study conducted in Ghana (17) and is lower than the study conducted in Bangladesh (37.8%) (39). The lower proportion might be the commencement time of the CBHI scheme. In Bangladesh, CBHI began a longer time ago than the present study which may create a good opportunity, change the attitude of the health workers over time, and create strong, conducive, and impressive health service delivery setups. Unlikely, our study finding revealed that the clients were mostly dissatisfied with the availability of the prescribed drug and requested lab tests; this was supported by a study conducted in Turkey (20). However, this finding was lower compared with a study conducted in Pakistan (96%). The higher achievement of the Pakistan study is possibly attributed to the area where the study was conducted that was in an urban setup and may improve the supply of logistics more than our study area. Availability of logistics came with affording ordered drugs as well as requested tests that may lead to increased satisfaction of the client.

The finding of this study is contradictory in terms of participant address with the study conducted in Turkey (20): 64.0% of the urban respondents were more satisfied than the rural respondents, whereas in our study, the rural residents were 1.8 times more likely satisfied than the urban residents. The possible reason might be the service charge-free system being new for the rural community, and they become satisfied with slight services compared with the urban inhabitants. The study observed that the clients who are more likely to visit healthcare facilities tend to be satisfied. Visiting HFs four to seven times for medical advice 13 times increases the satisfaction level of the clients; the finding can be supported by a study conducted in Zaria, Nigeria (42).

The study revealed that those within the age range between 35 and 44 years were 89.4% times less likely to be satisfied than their counterparts which is similar to the study in Turkey (20). When the age of the respondents is more advanced, they tend to be satisfied with slight services because their expectation and perception may not be as high as the middle- and young-aged participants.

## Conclusion and recommendation

The study finding revealed that the overall satisfaction level toward health service provision among the members of the CBHI scheme was high compared with the previous studies.

Our study found that age, residence, frequency of visiting the HFs, payment on the occasion of the referral services, level of knowledge of the client, and the size of the family were identified as predictors of client satisfaction.

Therefore, to improve enrollee satisfaction and client retention in the scheme health program manager, the respective facilities’ office chief executives should exhaustively engage on the availing laboratory test materials and drugs within the respective HFs and should have revised scheme issues as well as service delivery system for urban inhabitants.

The referral linkage from PHCU to the next-step HFs should be taken into account to avoid payment for service by Zonal and Woreda health offices through preparing a memorandum of understanding (MOU) between them.

## Data Availability

The original contributions presented in the study are included in the article/Supplementary Material, further inquiries can be directed to the corresponding author.
