# Supplementary material for: Client satisfaction and associated factors towards the health service provided to members of a community-based health insurance scheme in Southern Ethiopia
Source: Front Health Serv. 2023 Nov 3;3:1237895. doi: 10.3389/frhs.2023.1237895 (PMC10656742; doi:10.3389/frhs.2023.1237895)
Supplement: Supplementary file 1 [file Datasheet1.docx]

### Annexes

**Annex I**: **English version participants information sheet and consent form**

Title: enrolee’s satisfaction with health service provision under a health insurance scheme and the factors which influence the satisfaction in Silte zone, Hulberage woreda, SNNPR, Ethiopia, 2019.

Address: Phone (mob.) (251) 0923406980/0913791113

Email: [gossabo2004@gmail.com](mailto:gossabo2004@gmail.com)

Co-investigators: Teshome Tesfaye

Address: Phone^2^ +251 0913637771

Name of the organization: Wachemo University, School of Medicine & Health Science

Source of fund: WCU

**Information to study participants**

Greeting --------------------------------------------------------

My name is------------------------- I am instructor in Wachemo University. I am planning to conduct my study on to determine enrolee’s satisfaction with health service provision under a health insurance scheme and the factors which influence the satisfaction in Silte zone Hulberage woreda, SNNPR, Ethiopia, 2019. I am going to ask you some questions that are not difficult to answer. Whatever information you provide will be kept confidentiality. Your name will not be written in this format, never be used in connection with any of the information you are going to tell me and your name is not identified in any of this output. You are not obliged to answer any question that you do not want to answer and you may drop-out this interview at any time you want to. However, your honest answers to these questions will help us in identifying the determinant factors of pregnancy induced hypertension in the future.

We would appreciate your help in responding to these questions and we value your input to make this study a successful one.

| code | Questionnaire | | Response | | | | | Skip | |
| --- | --- | --- | --- | --- | --- | --- | --- | --- | --- |
|  | **Part I: Socio-demographic factors** | | | | | | |  | |
| 101 | Age of the respondents (HHs head) | 1. ------ | | | | | |  | |
| 102 | Sex | 1. Male 2. Female | | | | | |  | |
| 103 | Residence | 1. Rural 2. Urban | | | | | |  | |
| 103 | Educational status of respondents | 1. unable to write & read 2. able to read and write 3. 1-8^th^ 4. 9^th^ – 12^th^ 5. College & above | | | | | |  | |
| 105 | Occupation | 1. Students 2. Daily labor 3. Merchant/self business 4. Household wife 5. Farmer | | | | | |  | |
| 106 | Marital status | 1. single 2. married 3. divorced 4. widowed | | | | | |  | |
| 107 | Monthly income per month | 1. < 500 ETB 2. 500- 1000 ETB 3. > 1000 ETB | | | | | |  | |
| 108 | How long being member of CBHI | 1. One year 2. More than one year | | | | | |  | |
| 109 | Have you ever participated in ‘’ iddir’’ | 1. Yes 2. No | | | | | |  | |
| 110 | How many Birr have you been paying per/month per individual | ----- | | | | | |  | |
| 111 | Number of family included under HHs insurance scheme | ------ | | | | | |  | |
|  | **Part II: Knowledge on CBHI** | | | | | | |  | |
| 201 | Where did you heard about CBHI? | 1. HEWs 2. Other health professionals 3. Neighbours 4. Mass media 5. Kebele administrative | | | | | |  | |
| 202 | Do you know family members enrolled insured scheme (under HHs) | 1. Yes 2. No | | | | | |  | |
| 203 | Community members could be included in CBHI are | 1. Poor 2. Reach 3. Farmer 4. Merchant 5. Any one | | | | | |  | |
| 204 | Do you know after how many month could you get service following registration | 1. Yes 2. No | | | | | |  | |
| 205 | Do you know under HHs head scheme who can be enrolled | 1. Yes 2. No | | | | | | to Q # 207 | |
| 206 | If you said ‘’ yes’’ for the question # 205 who has mandate to be enrolled on CBHI | 1. Parents 2. All family members 3. Children only 4. Age bellow 18 yrs | | | | | |  | |
| 207 | How many time did you paid membership contribution/premium/ per year (schedule) | 1. Once 2. Twice 3. Three times 4. Four times | | | | | |  | |
| 208 | Do you know monthly payment per individual | 1. Yes 2. No | | | | | |  | |
| 209 | If you question is yes for Q # 208 what is its amount | --------- | | | | | |  | |
| 210 | How you perceive annual payment | 1. Expensive 2. Cheap 3. Very cheap | | | | | |  | |
| 211 | Being CBHI member is beneficiary | 1.yes  2. no | | | | | |  | |
| 212 | Do you know Service obtained are more than payment | 1. Yes 2. No | | | | | |  | |
| 213 | CBHI is non-profit for government which is stand serve for community | 1. Yes 2. No | | | | | |  | |
| 214 | Delaines of quarter or monthly payment will not discontinue service | 1. Yes 2. No | | | | | |  | |
| 215 | Participating and being member of CBHI is individual or HHs responsibilities | 1. Yes 2. No | | | | | |  | |
|  | **Part III:- Health service utilization** | | | | | | |  | |
| 301 | Have you illness encountered during the last 12 months | | 1. Yes 2. No | | | | |  | |
| 302 | How many time did you visit public health facility | | -------------- | | | | |  | |
| 303 | Have you ever participated in CBHI issue meeting | | 1. Yes 2. No | | | | |  | |
| 304 | Have you ever participated in a CBHI related training | | 1. Yes 2. No | | | | |  | |
| 305 | How you were enrolled in the CBHI | | 1. Voluntarily 2. Forced | | | | |  | |
| 306 | How much time it takes from nearest HFs | | 1. Less than 30 minute 2. 30 – 60 minute 3. More than an hour | | | | |  | |
| 307 | Have you ever referred to next further facility (hospital) for Dx and Rx | | 1. Yes 2. No | | | | |  | |
| 308 | Where did you referred | | 1. HC 2. Hospital | | | | |  | |
| 309 | If your answer is yes for # question --- , did you paid there for service and drug | | 1. Yes 2. No | | | | |  | |
| 310 | How many Birr you have paid | | ------- | | | | |  | |
| 311 | How about referral service | | 1. Very good 2. Good 3. Not too much good 4. Bad | | | | |  | |
|  | **Part IV:- clients satisfaction on health service delivery with CBHI scheme** | | | | | | | | |
|  |  | | | S.agree | Agree | neutral | Disagree | | S.disagree |
|  |  |  |  |  |  |  |  | |  |
| 401 | Obtaining Service is affordable with in the 24 hours | | |  |  |  |  | |  |
| 402 | Health care providers intimacy is like as friend for clients | | |  |  |  |  | |  |
| 403 | Health care providers can provide sufficient information for service | | |  |  |  |  | |  |
| 404 | Health care providers provides enough information about confidentiality | | |  |  |  |  | |  |
| 405 | Service delivery areas and set –ups are comfortable | | |  |  |  |  | |  |
| 406 | Enrolment in CBHI helps to get better health service | | |  |  |  |  | |  |
| 407 | Enrolment increases service utilization | | |  |  |  |  | |  |
| 408 | Service have been obtaining are greater than payment/premium | | |  |  |  |  | |  |
| 409 | Being membership decrease cost expenditure for medical bill | | |  |  |  |  | |  |
| 410 | CBHI office opening time is comfortable to obtain service | | |  |  |  |  | |  |
| 411 | All laboratory requested are available in the facility | | |  |  |  |  | |  |
| 412 | All prescribed drugs available in dispensary/pharmacy | | |  |  |  |  | |  |
| 413 | Time between HFs visit and service obtaining are average | | |  |  |  |  | |  |
| 414 | Health care providers can provide adequate time for exam and Rx | | |  |  |  |  | |  |
| 415 | HWS delivers service respectfully | | |  |  |  |  | |  |
| 416 | Membership registration time and service started time comfortable | | |  |  |  |  | |  |
|  | **Part V: Factors inhibit clients satisfaction** | | | | | | | | |
| 501 | Why not satisfied on CBHI scheme | 1. Unaware of insurance 2. Lack of adequate info. About CBHI 3. HFS had no adequate services 4. Low government concern 5. Lac of access of health service 6. Health facility is too far 7. Private institution is better than public 8. Others | | | | | | | |

**Thank you!**
